# Obituary

**Published:** 1873-04

**Authors:** R. E. McVey

**Affiliations:** Waverly, Ill.


### OBITUARY.

Dr. WILLIAM HENRY PALMER, the subject of this memoir, was born in Morgan County, Illinois, December 24th, 1849, and died of small-pox, at Leroy, Ill., March 5th, 1873.

Dr. Palmer leaves a mother and five brothers to mourn his loss. The oldest of his brothers is Gen. Joseph B. Palmer, of Murfreesboro', Tenn., whose name was before the constitutional convention of his district for Congressional honors, but withdrawn in favor of John M. Bright, the present incumbent.

In 1868, Dr. Palmer selected medicine as his profession and entered upon his studies with Dr. R. E. McVey, at Waverly, Ill., where he remained two years in close application to study, giving especial attention to the study of anatomy. Dr. Palmer's genial disposition and insinuating manner made him socially more attractive, and secured for him the universal respect of all with whom he associated, and the love of his intimate friends. He was, indeed, a young man of true merit and of unblemished character.

He was a member of the M. E. Church at Waverly, Ill., and was noted for his devotion to the interests of christianity and the church.

In the fall of 1869–70 he attended his first course of lectures in Rush Medical College. On account of the great fire he was prevented from completing his course of studies in the College until 1873. Few men ever give promise of a more useful and honorable life than did Dr. Palmer. *Requiescat in pace.*

R. E. McVEY.

Waverly, Ill., March 25, 1873.

